# Ginseng-derived exosomes loaded in fibrin gel promote retinal ganglion cell survival in glaucoma by exerting anti-inflammatory effects through modulating microglial polarization

**DOI:** 10.1093/rb/rbag146

**Published:** 2026-07-10

**Authors:** Dengming Zhou, Dehong Tan, Xuanqi Peng, Cailian Fang, Yongzhen Yu, Haroon Iqbal, Junfan Zhang, Lin Fu, Lewei Tang, Xiaoyu Zhou, Xinyue Zhang, Run Xiao, Wenxiang Zhu, Ludan Yue, Yuanbo Liang

**Affiliations:** State Key Laboratory of Ophthalmology, Optometry and Visual Science, Eye Hospital, Wenzhou Medical University, Wenzhou 325027, China; Eye Research Center, Hangzhou Institute of Medicine, Chinese Academy of Sciences, Eye Hospital, Wenzhou Medical University, Hangzhou 310018, China; Aier Academy of Ophthalmology, Central South University, Changsha 410083, China; Aier Glaucoma Institute, Changsha Glaucoma Diagnosis and Treatment Technology Innovation Center, Changsha Aier Eye Hospital, Changsha 410015, China; Department of Biochemistry and Molecular Biology, School of Medicine, Southeast University, Nanjing 210009, China; School of Molecular Medicine, Hangzhou Institute for Advanced Study, UCAS, Hangzhou 310024, China; State Key Laboratory of Ophthalmology, Optometry and Visual Science, Eye Hospital, Wenzhou Medical University, Wenzhou 325027, China; Eye Research Center, Hangzhou Institute of Medicine, Chinese Academy of Sciences, Eye Hospital, Wenzhou Medical University, Hangzhou 310018, China; State Key Laboratory of Ophthalmology, Optometry and Visual Science, Eye Hospital, Wenzhou Medical University, Wenzhou 325027, China; Eye Research Center, Hangzhou Institute of Medicine, Chinese Academy of Sciences, Eye Hospital, Wenzhou Medical University, Hangzhou 310018, China; State Key Laboratory of Ophthalmology, Optometry and Visual Science, Eye Hospital, Wenzhou Medical University, Wenzhou 325027, China; Eye Research Center, Hangzhou Institute of Medicine, Chinese Academy of Sciences, Eye Hospital, Wenzhou Medical University, Hangzhou 310018, China; State Key Laboratory of Ophthalmology, Optometry and Visual Science, Eye Hospital, Wenzhou Medical University, Wenzhou 325027, China; Eye Research Center, Hangzhou Institute of Medicine, Chinese Academy of Sciences, Eye Hospital, Wenzhou Medical University, Hangzhou 310018, China; State Key Laboratory of Ophthalmology, Optometry and Visual Science, Eye Hospital, Wenzhou Medical University, Wenzhou 325027, China; Eye Research Center, Hangzhou Institute of Medicine, Chinese Academy of Sciences, Eye Hospital, Wenzhou Medical University, Hangzhou 310018, China; Aier Academy of Ophthalmology, Central South University, Changsha 410083, China; Aier Glaucoma Institute, Changsha Glaucoma Diagnosis and Treatment Technology Innovation Center, Changsha Aier Eye Hospital, Changsha 410015, China; Aier Academy of Ophthalmology, Central South University, Changsha 410083, China; Aier Glaucoma Institute, Changsha Glaucoma Diagnosis and Treatment Technology Innovation Center, Changsha Aier Eye Hospital, Changsha 410015, China; School of Molecular Medicine, Hangzhou Institute for Advanced Study, UCAS, Hangzhou 310024, China; Aier Academy of Ophthalmology, Central South University, Changsha 410083, China; Aier Glaucoma Institute, Changsha Glaucoma Diagnosis and Treatment Technology Innovation Center, Changsha Aier Eye Hospital, Changsha 410015, China; Department of Biochemistry and Molecular Biology, School of Medicine, Southeast University, Nanjing 210009, China; State Key Laboratory of Ophthalmology, Optometry and Visual Science, Eye Hospital, Wenzhou Medical University, Wenzhou 325027, China; Eye Research Center, Hangzhou Institute of Medicine, Chinese Academy of Sciences, Eye Hospital, Wenzhou Medical University, Hangzhou 310018, China

**Keywords:** hydrogel, ginseng-derived exosomes, glaucoma, RGCs, microglia

## Abstract

Glaucoma is the leading cause of irreversible blindness globally. Chronic neuroinflammation drives progressive retinal ganglion cell (RGC) loss independent of intraocular pressure, yet safe, sustained and precise neuroprotective modulation of the retinal inflammatory microenvironment remains a significant challenge. We developed an injectable fibrin gel delivery system loaded with ginseng-derived exosomes (GE-fibrin gel, GE-gel) to modulate microglial polarization, reduce retinal inflammation and promote RGC survival. *In vitro*, GE showed strong antioxidant and anti-apoptotic effects by getting rid of reactive oxygen species caused by oxidative stress and lowering apoptosis in R28 cells. Both GE and GE-gel stopped lipopolysaccharide from causing pro-inflammatory M1 polarization of microglia and increased anti-inflammatory M2 phenotypic change. Intravitreal injection of GE-gel suppressed pro-inflammatory microglial activation, diminished neuroinflammation and improved the survival of RGCs in a chronic ocular hypertension rat model. These results show that GE-gel could promote microglial polarization, change the immune environment in the retina and protect RGCs functionally over the long term. This approach may hopefully provide promising solutions for rapid and effective glaucoma therapy.

## Graphical Abstract

**Graphical Abstract:**
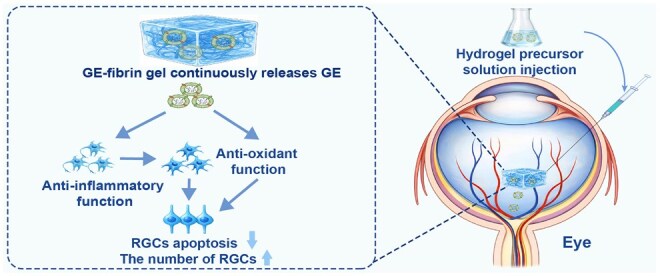

## Introduction

Glaucoma is the leading cause of irreversible blindness worldwide, characterized by progressive loss of retinal ganglion cells (RGCs) and degenerative damage to optic nerve axons, ultimately resulting in permanent visual impairment [1, 2]. Although elevated intraocular pressure (IOP) is widely recognized as the most important risk factor and the primary target of clinical intervention, substantial clinical and preclinical evidence indicates that a considerable proportion of patients continue to experience visual field deterioration despite effective IOP control [3, 4]. This suggests that, in addition to mechanical stress, IOP-independent neurodegenerative mechanisms play a key role in the onset and progression of glaucoma [5, 6]. Recent studies have highlighted neuroinflammation as a central pathological process driving RGC damage and disease progression [7]. Chronic low-grade retinal inflammation in glaucoma activates microglia, recruits peripheral immune cells and induces sustained release of pro-inflammatory cytokines, forming a vicious cycle of inflammation-oxidative stress-cell death that exacerbates neuronal injury [8]. As resident immune cells in the retina, microglia play a dual role in maintaining retinal homeostasis and responding to stress and their dynamic functional state critically influences neuronal fate [9, 10]. Under pathological stimuli such as elevated IOP, ischemia, hypoxia or metabolic stress, microglia polarize from a resting state toward the pro-inflammatory M1 phenotype, releasing tumor necrosis factor-α (TNF-α), interleukin-1β (IL-1β), inducible nitric oxide synthase and excessive reactive oxygen species (ROS), directly inducing RGC apoptosis [11, 12]. Importantly, even after IOP normalization, persistently activated M1 microglia maintain an inflammatory environment, driving irreversible neurodegeneration [13, 14]. Therefore, targeted modulation of microglial polarization has been considered a promising strategy for neuroprotection in glaucoma.

Plant-derived exosomes have recently emerged as natural nanovesicles with broad therapeutic potential due to their wide availability, excellent biocompatibility, relatively low production cost and capacity to carry diverse bioactive molecules, including nucleic acids, proteins, lipids and plant-specific metabolites [15, 16]. Compared with animal-derived exosomes, plant exosomes offer a lower risk of viral contamination, reduced immunogenicity and greater scalability [17, 18]. Previous studies have demonstrated the anti-inflammatory, antioxidative, immunomodulatory and tissue-regenerative effects of various plant-derived exosomes [19, 20]. For instance, engineered *Pueraria* exosomes achieve brain-targeted delivery, improving mitochondrial function in Parkinson’s disease models via PINK1-Parkin-mediated mitophagy [15], Gouqi berry-derived nanovesicles promote osteogenic differentiation through PI3K/Akt signaling to accelerate fracture repair [21] and honeysuckle-derived exosome-like vesicles ameliorate metabolic-associated fatty liver disease by restoring gut barrier integrity and regulating microbiota metabolism [22]. These studies collectively highlight the broad potential of plant exosomes in immune microenvironment modulation and tissue repair.

Ginseng (Panax ginseng), a traditional medicinal herb, contains active compounds such as ginsenosides, polysaccharides and miRNAs known for their anti-inflammatory, antioxidative and immunomodulatory properties [23]. Ginseng-derived exosomes (GE) have membranes that stay intact and a lot of bioactive cargo. Previous research indicates that GE diminishes oxidative stress and apoptosis by inhibiting MAPK signaling, thereby alleviating chemotherapy-induced cardiotoxicity [24]. Additionally, functionalized GE embedded in hydrogels can promote angiogenesis and tissue repair [25]. Ginseng-derived nanoparticles can induce M2 macrophage polarization via the PI3K/AKT/HIF-1α pathway, facilitating wound healing in diabetic models [26]. These findings suggest that GE may possess the capacity to alter immunological microenvironments by promoting anti-inflammatory phenotypic conversion. However, direct intraocular administration of free exosomes faces several clinical challenges, including rapid clearance by the vitreous humor and aqueous outflow pathways, short intraocular retention time, limited bioavailability at the target site and potential off-target effects. These barriers hinder sustained therapeutic efficacy and necessitate repeated injections, which increase the risk of endophthalmitis and retinal detachment.

**Figure 1:**
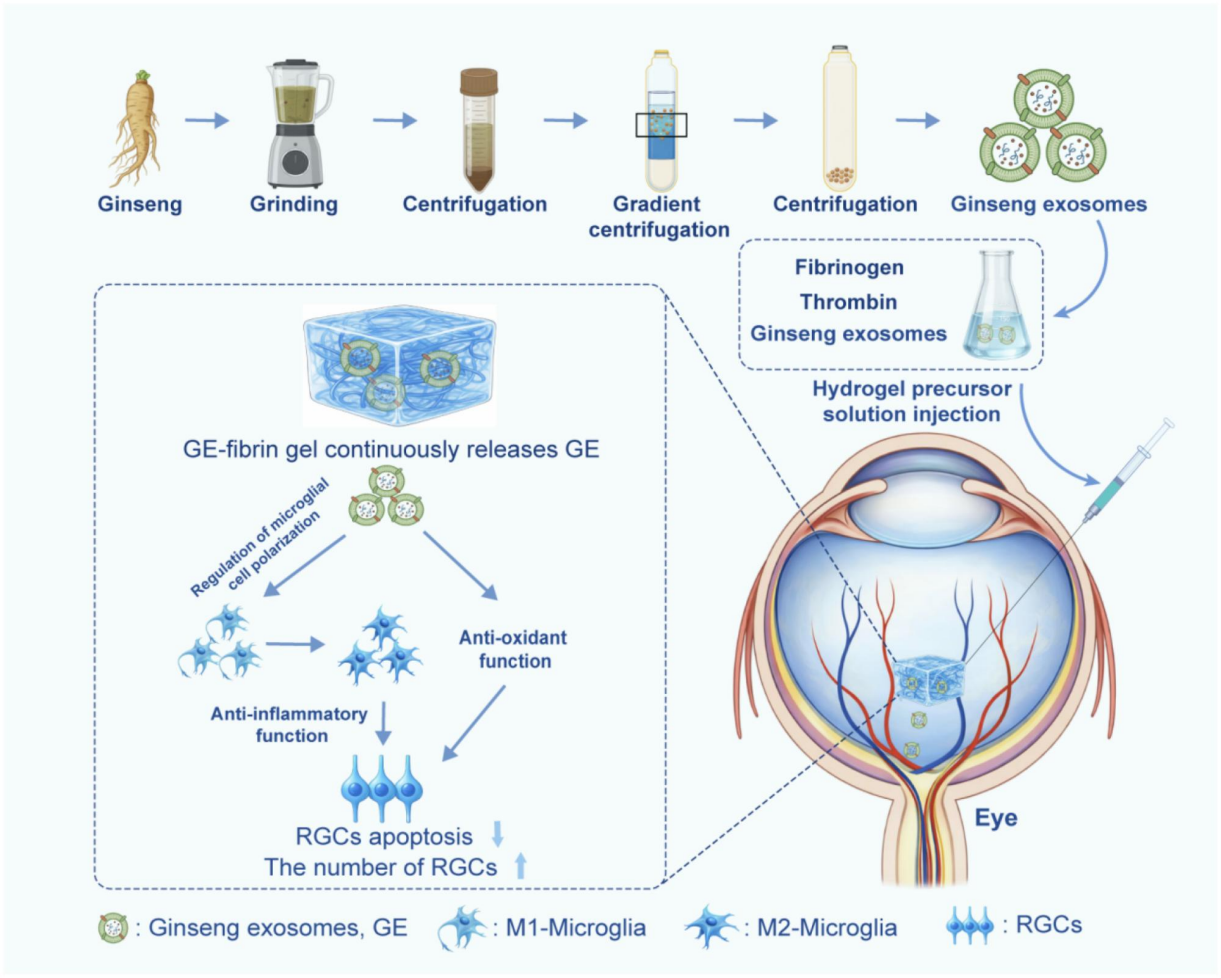
Schematic illustration of an injectable fibrin-based hydrogel delivery system loaded with GE for the precise modulation of glaucoma-associated neuroinflammation and protection of RGCs.

Fibrin gel, a Food and Drug Administration-approved natural biomaterial, offers excellent biocompatibility, injectability and biodegradability. Its 3D network can serve as the depot for localized and sustained release of therapeutic agents [27, 28]. Unlike synthetic polymers, fibrin gel mimics the native extracellular matrix and avoids toxic crosslinkers or organic solvents, making it particularly suitable for delicate intraocular tissues. Integrating exosomes within the fibrin matrix, therefore, represents an attractive strategy to improve their intraocular retention and bioactivity. Based on this concept, we constructed an injectable fibrin gel encapsulating GE (GE-gel) to regulate microglial polarization, suppress retinal neuroinflammation and support long-term survival and function of RGCs in glaucoma (Figure 1). The therapeutic performance of GE-gel was systematically evaluated through *in vitro* assays and in a chronic ocular hypertension (COH) rat model. Multimodal ophthalmic imaging, electrophysiological assessment, transcriptomic profiling and single-cell RNA sequencing (scRNA-seq) were employed to elucidate both efficacy and the underlying molecular mechanisms.

## Materials and methods

### Materials and characterization

Fibrinogen, cell membrane fluorescent probe DiI and lipopolysaccharide (LPS) were purchased from Solarbio Technology Co., Ltd. (Beijing, China). Thrombin was obtained from McLean Biochemical Technology Co., Ltd. (Shanghai, China). Ginseng roots were sourced from a cooperative farm in Hunan Province, China. Calcein-AM/PI dual-staining kit was obtained from Yisheng Biotechnology Co., Ltd. (Shanghai, China). DAPI staining solution was purchased from Meilun Biotechnology Co., Ltd. (Dalian, China). ROS detection kits (Dichlorodihydrofluorescein diacetate (DCFH-DA) and dihydroethidium (DHE)) were obtained from Biyuntian Biotechnology Co., Ltd. (China). Flow cytometry antibodies CD86-FITC and CD206-APC were purchased from Thermo Fisher Scientific (China). The bicinchoninic acid assay (BCA) kit was purchased from Yamei Biopharmaceutical Technology Co., Ltd. (Shanghai, China). Other chemical reagents were purchased from China National Pharmaceutical Group Chemical Reagents Co., Ltd. Male Sprague–Dawley rats (6–8 weeks old) were obtained from Slack Jingda Laboratory Animal Co., Ltd. (Hunan, China). All animal experiments were approved by Aier Eye Hospital Laboratory Animal Welfare and Ethics (Protocol number: AEI2025020).

### Preparation and characterization of GE

Ginseng roots were cleaned, chopped and homogenized using a juicer to obtain crude ginseng juice. The juice was sequentially centrifuged at 200 × g for 10 min, 2000 × g for 20 min and 10000 × g for 30 min to remove whole cells and debris. The supernatant was filtered through a 0.45 μm membrane and then ultracentrifuged at 100 000 × g for 60 min. The resulting pellet was resuspended in PBS and further purified by sucrose density gradient centrifugation (15%, 30%, 45% and 60% w/v). The band at the 30%/45% interface, containing high-purity GE, was collected and subjected to a final ultracentrifugation at 100 000 × g for 60 min to remove residual sucrose. Purified GE were resuspended in PBS and stored at −80°C.

The total protein content of GE was determined using a BCA protein assay kit. Morphological characterization was performed by transmission electron microscopy (TEM) (JEM-2100 Plus, JEOL, Japan). Hydrodynamic diameter and zeta potential were measured using dynamic light scattering (DLS) (Zetasizer Nano ZS, Malvern, UK) and nanoparticle tracking analysis (NTA) (ZetaView PMX 110, Particle Metrix, Germany), respectively.

### Preparation and characterization of GE-gel

Following published methods for exosome-loaded fibrin gels [29], GE was incorporated into fibrin gels. Fibrinogen solution (40 mg/mL in PBS) was mixed with GE solution (120 μg/mL in PBS) at a 1:1 volume ratio. The mixture was then combined with an equal volume of thrombin solution, rapidly forming a stable GE-loaded hydrogel (GE-gel) with a final GE concentration of 30 μg/mL. Microstructural differences between blank fibrin gel and GE-gel were assessed using scanning electron microscopy (SEM, HITACHI S4800, Japan).

### *In vitro* cellular uptake of GE

GE was fluorescently labeled with DiI by incubating GE (∼30 μg/mL) with DiI (10 μM) at 37°C for 30 min. R28 cells were placed in confocal culture dishes at a density of 1 × 10^6^ cells per well and grown for 12 h. Then, GE-DiI (30 μg/mL) was added, and the cells were allowed to grow for 1.5, 3, 6 or 12 h. After incubation, the media were taken away, and the cells were washed three times with PBS. The cells were then fixed with 4% paraformaldehyde at room temperature for 15 min. After that, they were washed three more times with PBS and stained with DAPI to visualize the nuclei. After three further PBS washes, a laser scanning confocal microscope was used to obtain fluorescence images of the cells to evalute how well they took up the substance.

### Establishment of H_2_O_2_-induced oxidative stress model in R28 cells

Cell viability under oxidative stress was assessed using the CCK-8 assay. R28 cells were seeded at a density of 5 × 10^3^ cells per well in 96-well plates and allowed to adhere. After attachment, cells were exposed to H_2_O_2_ at final concentrations of 0, 30, 60, 90, 120 or 150 μM for 24 h. Subsequently, CCK-8 solution was added according to the manufacturer’s protocol, and absorbance at 450 nm was measured using a microplate reader to quantify viable cells.

### *In vitro* live/dead cell staining

R28 cells were seeded at 1 × 10^5^ cells per well in 48-well plates and allowed to adhere. Cells were divided into four experimental groups: (i) control, no treatment, (ii) H_2_O_2_, treated with 90 μM H_2_O_2_, (iii) GE, treated with 30 μg/mL GE in combination with 90 μM H_2_O_2_ and (iv) GE-gel, exposed to 90 μM H_2_O_2_ in the lower chamber of a Transwell system with GE-gel placed in the upper chamber. After 24 h, the medium was removed and cells were incubated with Calcein-AM/PI staining solution at 37°C for 15 min in the dark. Following gentle PBS washes, fluorescence images were acquired using an inverted microscope. Viable cells exhibited green fluorescence (Calcein-AM), whereas dead cells showed red fluorescence (PI), allowing assessment of cell survival via dual-channel imaging.

### *In vitro* apoptosis assay

We used Annexin V-FITC/PI double labeling to assess apoptosis in the four treatment groups. In short, cells were put back into binding buffer, treated with Annexin V-FITC and PI at room temperature for 15 min in the dark and then evaluated by flow cytometry. We used FlowJo software (Version X, Stanford University) to process the data and count the number of cells that were dying early and late.

### Intracellular ROS measurement

To assess oxidative stress-induced ROS accumulation, R28 cells from identical treatment groups were treated with the fluorescent probes DCFH-DA (10 μM) or DHE (10 μM) at 37°C for 30 min in the dark. Following PBS washing, fluorescence intensity was examined using a laser scanning confocal microscope. Oxidized DCFH-DA gave off green fluorescence, and DHE bound to superoxide anions gave off red fluorescence, showing the ROS level in the cells.

### BV2 microglial polarization assay

We put 1 × 10^5^ BV2 cells in each of the six wells of a 24-well plate and let them grow overnight. After that, the cells were put in serum-free media to keep serum from affecting LPS activity. They were then treated with 1 μg/mL LPS for 12 h to cause M1 polarization. Next, the cells were split into three groups: (i) LPS (only LPS stimulation), (ii) GE (LPS stimulation followed by treatment with 30 μg/mL GE) and (iii) GE-gel (LPS stimulation followed by co-culture with GE-gel in a Transwell system). For Transwell co-culture experiments, GE-gel was placed in the upper chamber of a 24-well Transwell insert (Corning, polycarbonate membrane). BV2 cells were seeded in the lower chamber. This setup allowed diffusion of soluble factors and controlled release of GE from the gel without direct cell-gel contact. The Transwell inserts were pre-equilibrated with culture medium for 1 h before use. After 24 h, the cells were collected and stained with anti-CD86-FITC and anti-CD206-APC antibodies. We used flow cytometry to count the M1 (CD86^+^) and M2 (CD206^+^) populations to evaluate how different treatments affected the polarization of microglia.

### *In vivo* animal experiments

We made a COH model in Sprague–Dawley rats. We injected hyperosmotic saline into the scleral vein. This damages the endothelium and causes fibrosis in the episcleral veins. IOP stays high for >4 weeks. First, the rats were anesthetized. Then, a microinjection pump was used to inject hyperosmotic saline into their episcleral veins. After the needle was removed, antibiotic ointment was applied to the area. Animals were randomly assigned to four groups: (i) normal control (NC): no treatment, (ii) COH model: model induction only, (iii) GE treatment: intravitreal injection of 5 μL GE solution (30 μg/mL) immediately post-modeling (without anterior chamber paracentesis) and (iv) GE-gel treatment: intravitreal injection of 5 μL GE-gel containing an equivalent GE dose. Among them, both GE and GE-gel were administered as a single injection. IOP was monitored regularly. Four weeks post-intervention, animals were euthanized and eyes were collected for subsequent analyses.

#### Retinal morphology and immunofluorescence

Retinas were rapidly dissected into two portions. One portion was prepared as whole mounts and stained with anti-Brn3a antibody to quantify RGCs and another portion was fixed in 4% paraformaldehyde, paraffin-embedded and sectioned. Hematoxylin and eosin (H&E) staining evaluated retinal layer structure, and RNA-binding protein with multiple splicing (RBPMS) immunofluorescence further verified RGC survival.

#### Microglial analysis

Retinal sections were stained with IBA1 for immunofluorescence and observed under a laser scanning confocal microscope.

#### *In vivo* retinal imaging

Retinal structure was imaged *in vivo* using the Phoenix Micro IV system with an optical coherence tomography (OCT) module. Rats were anesthetized with Zoletil 50, and corneal anesthesia was achieved using 0.4% oxybuprocaine hydrochloride. Mydriasis was induced with tropicamide, and carbomer eye drops maintained corneal hydration. Images were captured under rat mode with 20–40 scans averaged per session for a high signal-to-noise ratio, alongside fundus photography.

#### Electroretinography

Retinal function was assessed using the Phoenix Ganzfeld Electroretinography (ERG) system. After dark adaptation for 8 h, responses to 3.1 log cd·s/m^2^ green light (505 nm, 5 ms) were recorded to evaluate photoreceptor and bipolar cell function (filter 2–1000 Hz, 300 ms recording, 50 averages). For light-adapted ERG, animals were exposed to 0.7 log cd·s/m^2^ background light for 10 min, followed by 4 log cd·s/m^2^ flashes to assess RGC function (filter 2–200 Hz, 1 s recording, 40 averages).

### Retinal RNA-Seq in rats

To elucidate the mechanism of GE-gel treatment, retinas from COH and GE-gel-treated rats were harvested at week 4 post-intervention, snap-frozen in liquid nitrogen and stored at −80°C. Total RNA was extracted, and library construction and sequencing were performed by Guangzhou Codio Biotechnology Co., Ltd. Differential expression analysis was conducted using the edgeR R package with thresholds of |log_2_FC| > log_2_(1.5) and adjusted *P* < 0.05. GO and KEGG pathway enrichment analyses were performed using the clusterProfiler package to identify key biological processes and signaling pathways.

### Retinal scRNA-seq

For cell-resolution analysis, retinas were digested into single-cell suspensions using the SeekMate Tissue Dissociation Kit A Pro (SeekGene, K01801301), followed by DNase I treatment and red blood cell removal. Cell viability (>85%) was assessed using the Countstar® Rigel S2 system with AO/PI staining. Cells were resuspended in RPMI 1640 medium containing 2% FBS. Single-cell 3′ libraries were prepared using the SeekOne® digital droplet system, following standard droplet generation, *in situ* reverse transcription, cDNA amplification and library purification protocols. Qualified libraries were sequenced on Illumina NovaSeq 6000 or DNBSEQ-T7 platforms with PE150 reads.

### Statistical analysis

Data are presented as mean ± SD with ≥3 biological replicates per group. Two-group comparisons used an unpaired two-tailed Student’s *t*-test, while multi-group comparisons used one-way or two-way ANOVA with Tukey’s *post hoc* test. Statistical analysis and figure generation were performed using GraphPad Prism. *P* < 0.05 was considered statistically significant, indicated as *P < 0.05, **P < 0.01, ***P < 0.001.

## Results and discussion

### Characterization of GE and GE-gel

The successful construction of a delivery system with both good biocompatibility and sustained-release capability is the basis for achieving the intended therapeutic function. Therefore, we first looked at the main physicochemical properties of GE taken from ginseng and their combination with GE-gel to verify whether they could be used as a sustained-release carrier.

**Figure 2:**
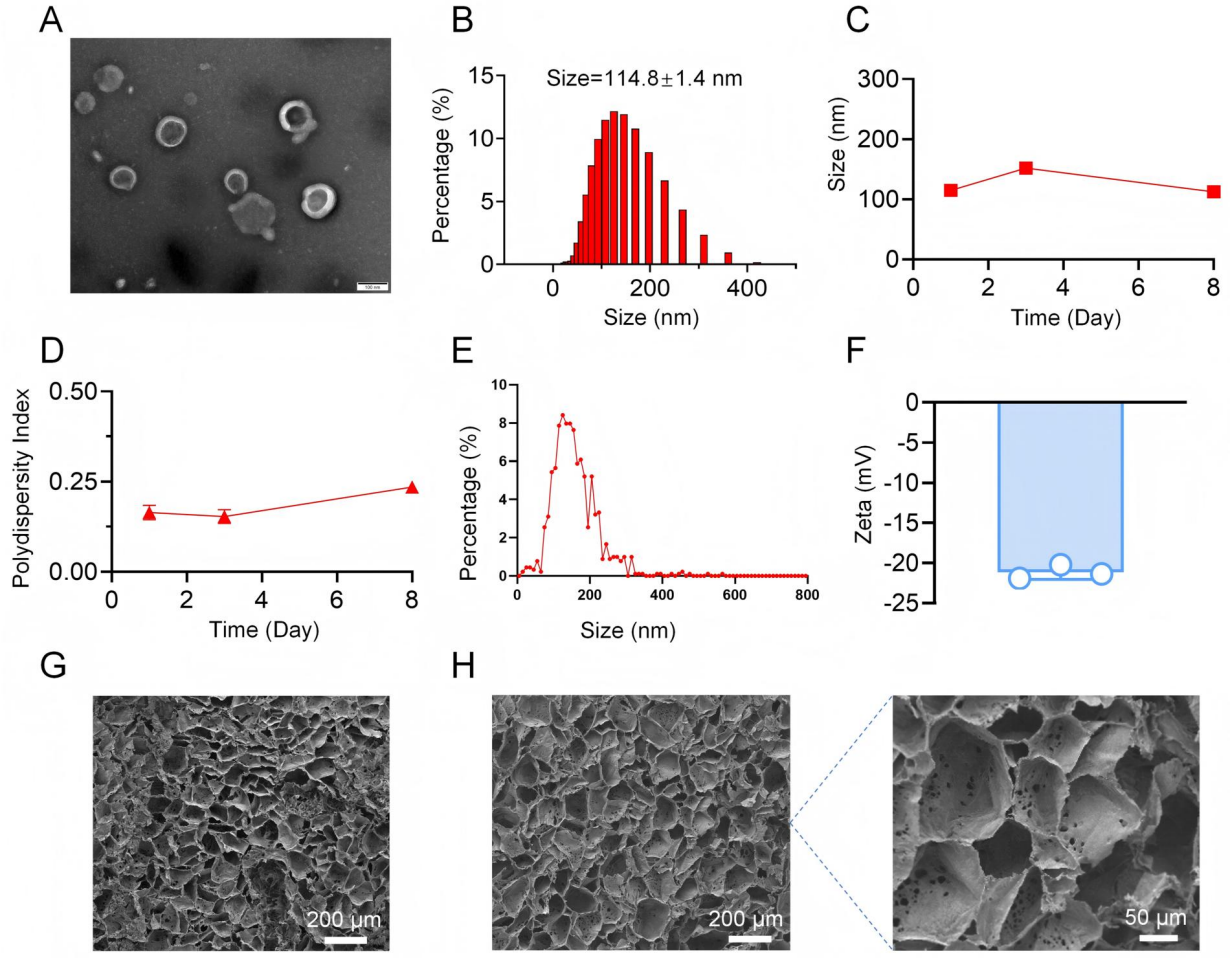
Characterization of GE and GE-gel. (**A**) TEM image of GE. (**B**) Hydrodynamic size distribution of GE. (**C**) Stability of GE. (**D**) PDI of GE. (**E**) Size distribution of GE measured by NTA. (**F**) Zeta potential of GE. SEM images of (**G**) fibrin gel and (**H**) GE-gel.

TEM was used to characterize the morphological features of the extracted GE. TEM images (Figure 2A) clearly showed that the particles we got had the typical cup-shaped or spherical shape and intact membrane structures, which are what extracellular vesicles look like at the ultrastructural level. DLS measurements indicated that the hydrodynamic diameter of GE was ∼114.8 ± 1.4 nm (Figure 2B), exhibiting a narrow size distribution, in accordance with TEM observations. Exosomes need to be stable in colloids for a long time to have long-lasting effects. To achieve this, the particle size and polydispersity index (PDI) of GE preserved in PBS at 4°C for 8 days were assessed. The results showed that the hydrodynamic diameter stayed the same over the observation period, with no major aggregation (Figure 2C) and the PDI stayed below 0.3 (Figure 2D) the whole time. Additionally, NTA was utilized to quantify particle concentration and size distribution in solution. The NTA profile displayed a singular principal peak (Figure 2E), corroborating that the predominant vesicles were concentrated within 100–200 nm, with an average diameter of roughly 125 nm. Zeta potential measurements showed that the surface charge was ∼−21.2 mV (Figure 2F), which was due to the phospholipid parts of the lipid bilayer. In addition, the CCK-8 assay revealed that R28 cells exhibited high cell viability when the concentration of GE was 30 μg/mL (Supplementary Figure S1).

We used SEM to look at the microstructure of the GE-gel. Figure 2G and H show that both the blank gel and the GE-gel made a 3D network with many connections and pores, between 50 and 150 μm. This porous structure lets nutrients and metabolic wastes pass through it easily [30]. At a higher magnification, the gel framework looked smooth, which suggested that GE might be well-encapsulated in the fibrin network or stuck to the pore walls. This kind of physical encapsulation allows GE to be released over time, making it the perfect carrier for future *in vitro* and *in vivo* functional research.

### *In vitro* cellular studies

We wanted to see how GE and GE-gel work when they are in situations that are similar to glaucoma’s. To start, we made cell models that look like the main types of damage that happen in the disease. These were oxidative stress and inflammation. After that, we looked at how GE and GE-gel affected neurons in the retina. We examined cell viability, apoptosis, intracellular ROS and the phenotypic transformation of microglia.

**Figure 3:**
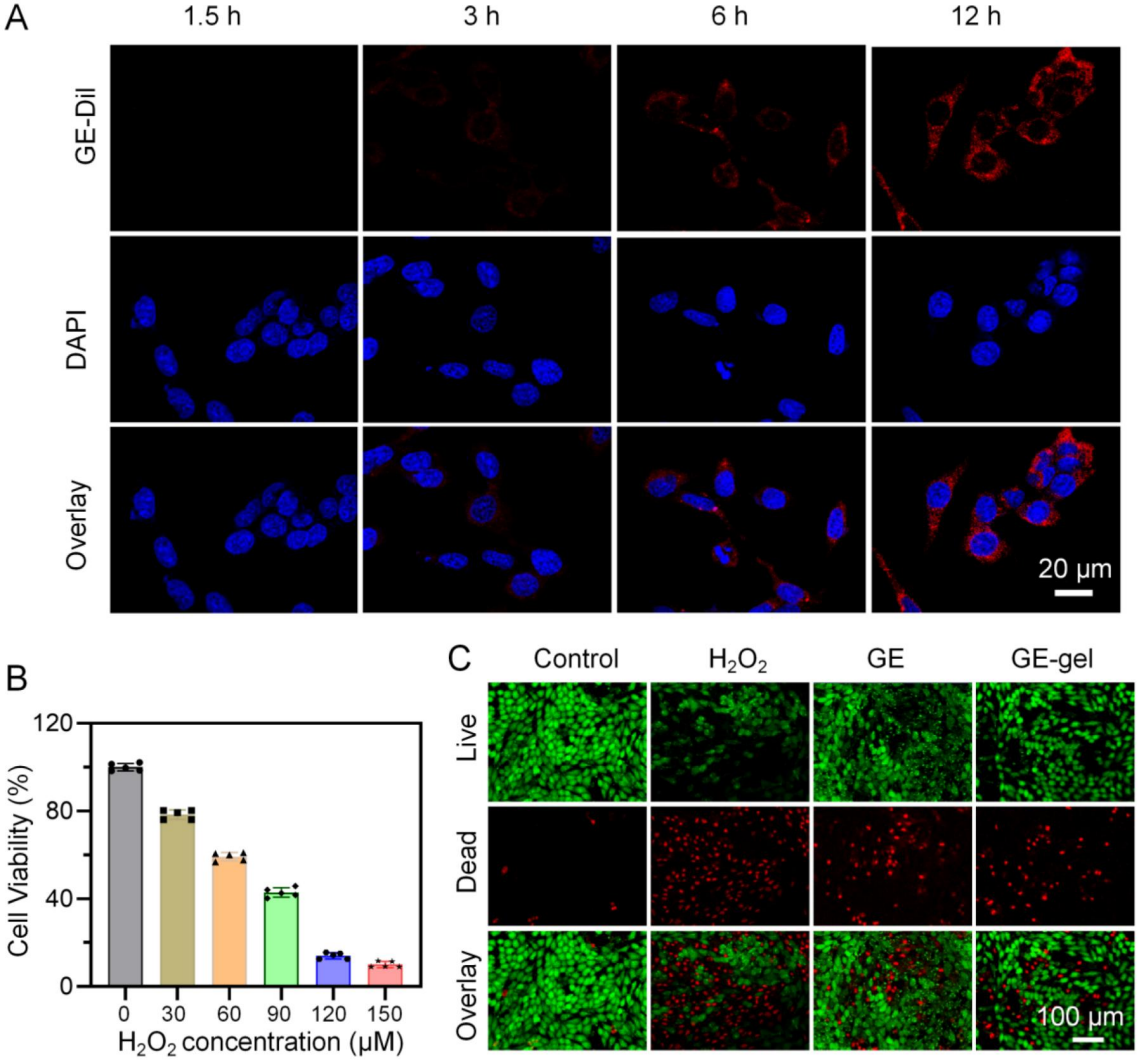
*In vitro* cellular evaluation of GE. (**A**) Fluorescence images showing uptake of DiI-labeled GE by R28 cells. (**B**) Cytotoxicity of R28 cells after H_2_O_2_ treatment. (**C**) Live/dead staining of R28 cells.

To determine whether GE could effectively interact with target cells, we first examined their cellular uptake in R28 cells. Confocal microscopy showed that DiI-labeled GE was internalized by the cells. The fluorescence signal gradually increased as the incubation time extended. After 12 h, clear fluorescence accumulation was observed around the nucleus (Figure 3A), indicating successful intracellular localization. These results suggest that GE can be efficiently taken up by R28 cells in a time-dependent manner. Such uptake provides the foundation for GE to influence intracellular biological processes and potentially exert neuroprotective effects.

Oxidative stress is a fundamental pathogenic component inducing RGC apoptosis in glaucoma [31]. We created a model of R28 cell damage caused by H_2_O_2_, and CCK-8 tests showed that the cells’ ability to live diminished in a dose-dependent way (Figure 3B). We used a concentration of 90 μM H_2_O_2_ to cause a lot of oxidative damage while yet keeping enough cells for testing the treatment. In live/dead staining studies (Figure 3C), both GE and GE-gel pretreatments significantly mitigated H_2_O_2_-induced cell death, preserving a substantial proportion of viable cells. The protective efficacy of the GE-gel group was comparable to that of free GE, demonstrating that encapsulation within the fibrin gel did not impact bioactivity.

**Figure 4:**
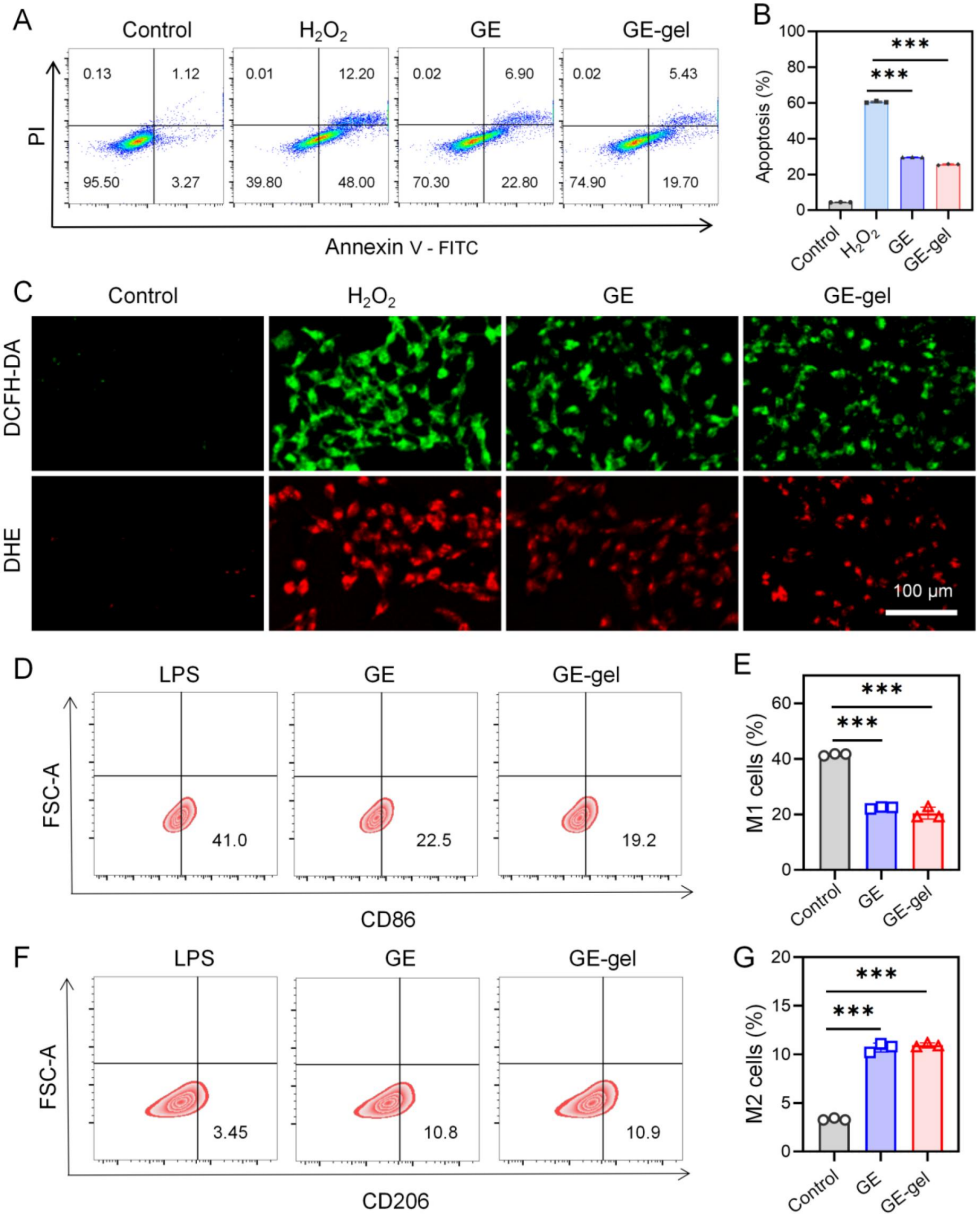
*In vitro* evaluation of GE on anti-apoptotic, antioxidative and immunomodulatory effects. (**A**) Flow cytometric analysis of apoptosis in R28 cells. (**B**) Quantification of apoptotic cell populations. (**C**) DCFH-DA and DHE fluorescence staining to assess intracellular ROS levels in R28 cells. (**D**) Flow cytometric analysis of CD86 expression (M1-polarization marker) in BV2 microglia. (**E**) Quantification of M1-polarized BV2 cells. (**F**) Flow cytometric analysis of CD206 expression (M2-polarization marker) in BV2 microglia. (**G**) Quantification of M2-polarized BV2 cells.

To further elucidate the protective mechanism, we assessed cell apoptosis and intracellular ROS levels. Flow cytometry analysis indicated that GE and GE-gel treatment significantly reduced H_2_O_2_-induced R28 cell apoptosis (Figure 4A and B). Fluorescence probe assays further demonstrated that both treatments effectively scavenged excessive intracellular ROS generated under H_2_O_2_ stimulation (Figure 4C). These results directly confirm the strong antioxidant and anti-apoptotic capacities of GE, consistent with known bioactive components of ginseng (such as ginsenosides and polysaccharides), which may alleviate oxidative stress either by directly neutralizing ROS or by activating endogenous cellular antioxidant defense systems, thereby blocking apoptosis signaling pathways and providing critical survival support for RGCs under pathological stress [32].

Microglia can cause inflammation and nerve cell damage that lasts in people with glaucoma [33]. We used BV2 cells to investigate how GE and GE-gel affect microglia. Flow cytometry showed that GE and GE-gel reduced the number of M1 microglia (CD86^+^) and increased the number of M2 microglia (CD206^+^) in cells that had been treated with LPS to cause inflammation (Figure 4D–G). This means that exosomes can change microglia from a harmful type to a helpful type. In glaucoma, this could help the retina by lowering harmful molecules like TNF-α and IL-1β and raising helpful growth factors. This can slow down the cycle of inflammation, oxidative stress and cell death, which helps RGCs stay alive. These results are consistent with previous research demonstrating that plant exosomes can alter immune cells [34, 35].

Taken together, the *in vitro* experiments comprehensively validated the therapeutic potential of the GE-gel system. It is effectively internalized by target cells and exerts protective effects through a multi-modal mechanism of ‘antioxidant-anti-apoptotic-immunomodulatory’ actions, targeting key pathological processes in glaucoma and providing a solid theoretical basis for its *in vivo* efficacy assessment.

### *In vivo* animal studies

To translate the *in vitro* observed antioxidant, anti-apoptotic and immunomodulatory potential of GE-gel into actual therapeutic efficacy, we systematically evaluated its protective effects on RGCs, retinal structural and functional improvements and modulation of the neuroinflammatory microenvironment in a COH rat model, which closely mimics clinical pathological conditions.

**Figure 5:**
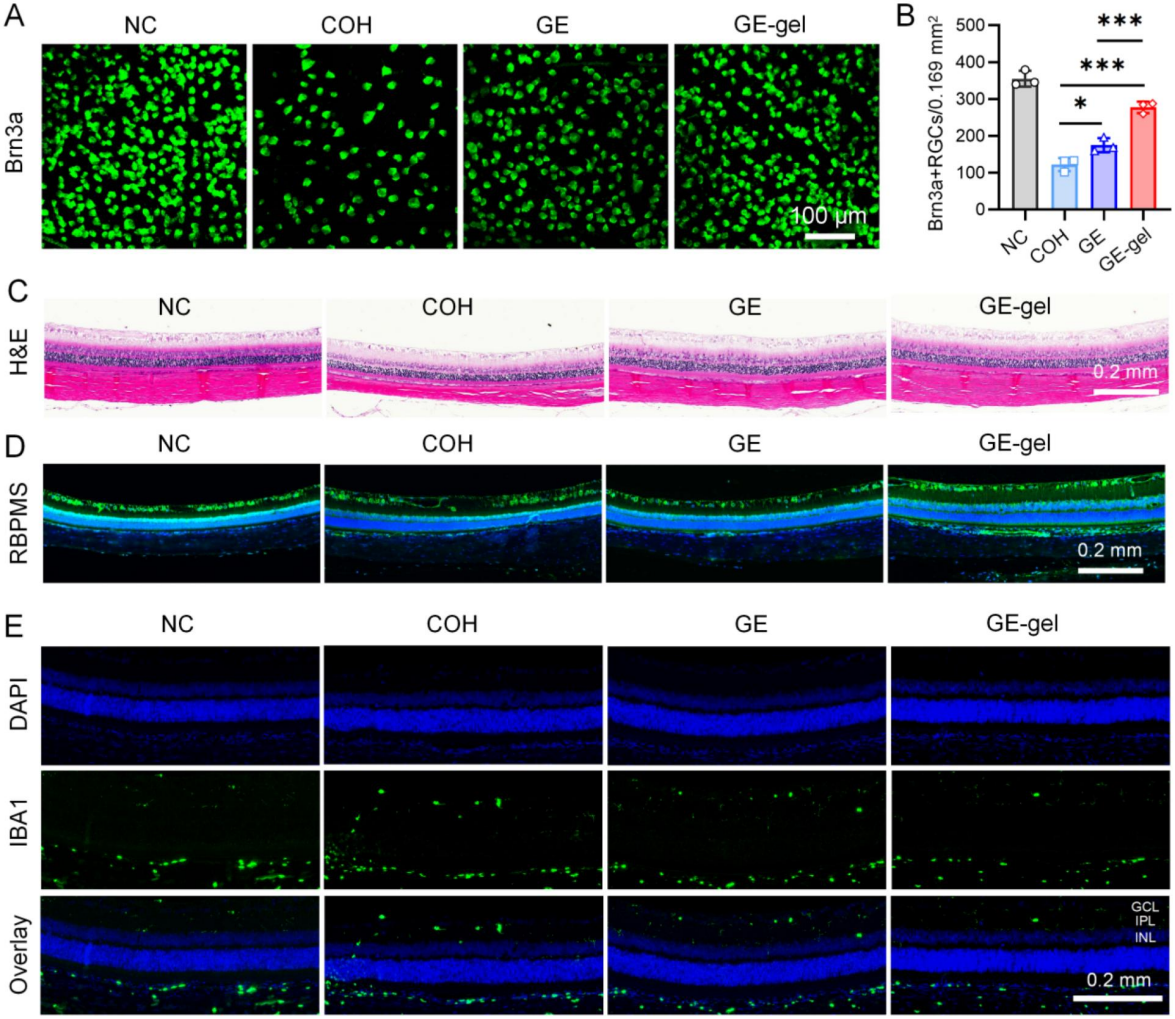
*In vivo* evaluation of GE and GE-gel treatment. (**A**) Immunofluorescence staining of Brn3a in whole-mount retinas to label RGCs. (**B**) Quantification of RGCs based on Brn3a-positive cells. (**C**) H&E staining and RBPMS immunofluorescence of rat retinal tissue. (**D**) Quantification of RGCs based on RBPMS-positive cells. (**E**) Immunofluorescence staining of IBA1 in rat retinas to assess microglial polarization.

The COH model in SD rats was established by injecting hypertonic saline into the scleral vein, resulting in endothelial cell damage and trabecular fibrosis, which elevated IOP. There were four groups of animals: NC, COH, GE and GE-gel. Supplementary Figure S2 shows that the IOP was higher in the COH, GE and GE-gel groups than in the NC group. This shows that persistent ocular hypertension was successfully brought on. In addition, we performed short-term IOP measurements following intravitreal injection of a 5 μL volume. As shown in Supplementary Figure S3, after injecting 5 μL of GE-gel into the rat vitreous cavity, the IOP transiently increased and then returned to normal levels within 5 min, indicating that a 5-μL injection volume has a minimal impact on IOP in rats. The primary pathogenic mechanism leading to vision loss in glaucoma is the apoptosis of RGCs. We checked whether RGCs were alive by using Brn3a immunofluorescence labeling on retinal flat mounts (Figure 5A). Quantitative analysis revealed a decrease in RGC density in the COH group compared with NC group, consistent with the neurodegenerative pathology of glaucoma. Both intravitreal injections of GE and GE-gel reduced RGC loss. The RGC density in the GE-gel group was greater than in the GE group (Figure 5B). Moreover, Dio dye labeling of GE in the GE and GE-gel groups (Supplementary Figures S4 and S5) demonstrated enhanced fluorescence in the retinal tissue of the GE-gel group at day 14, indicating that the hydrogel supports sustained retention of GE. This shows that the fibrin gel carrier is a long-lasting release platform that keeps the therapeutic exosome concentration in the eye for a long time.

Histological research corroborated these protective effects at the structural level. H&E staining (Figure 5C) and immunofluorescence labeling for the RGC-specific marker RBPMS (Figure 5D and Supplementary Figure S6) demonstrated notable thinning of the ganglion cell layer (GCL) and inner plexiform layer (IPL) in the COH group. The GE-gel treatment kept the normal thickness and structure of the retinal layers, and the number of RBPMS-positive cells got close to normal levels.

Neuroinflammation, especially abnormal microglial activation, is a significant contributor to RGC destruction in glaucoma. We employed immunofluorescence labeling to look at alterations in microglia in the retina (Figure 5E and Supplementary Figure S7). The COH group exhibited an elevated microglial count. Conversely, both GE and GE-gel treatments decreased microglial cell counts. These results suggest that GE-gel diminishes microglial cells, hence producing an anti-inflammatory impact, which may constitute a fundamental mechanism for its preservation of RGCs.

**Figure 6:**
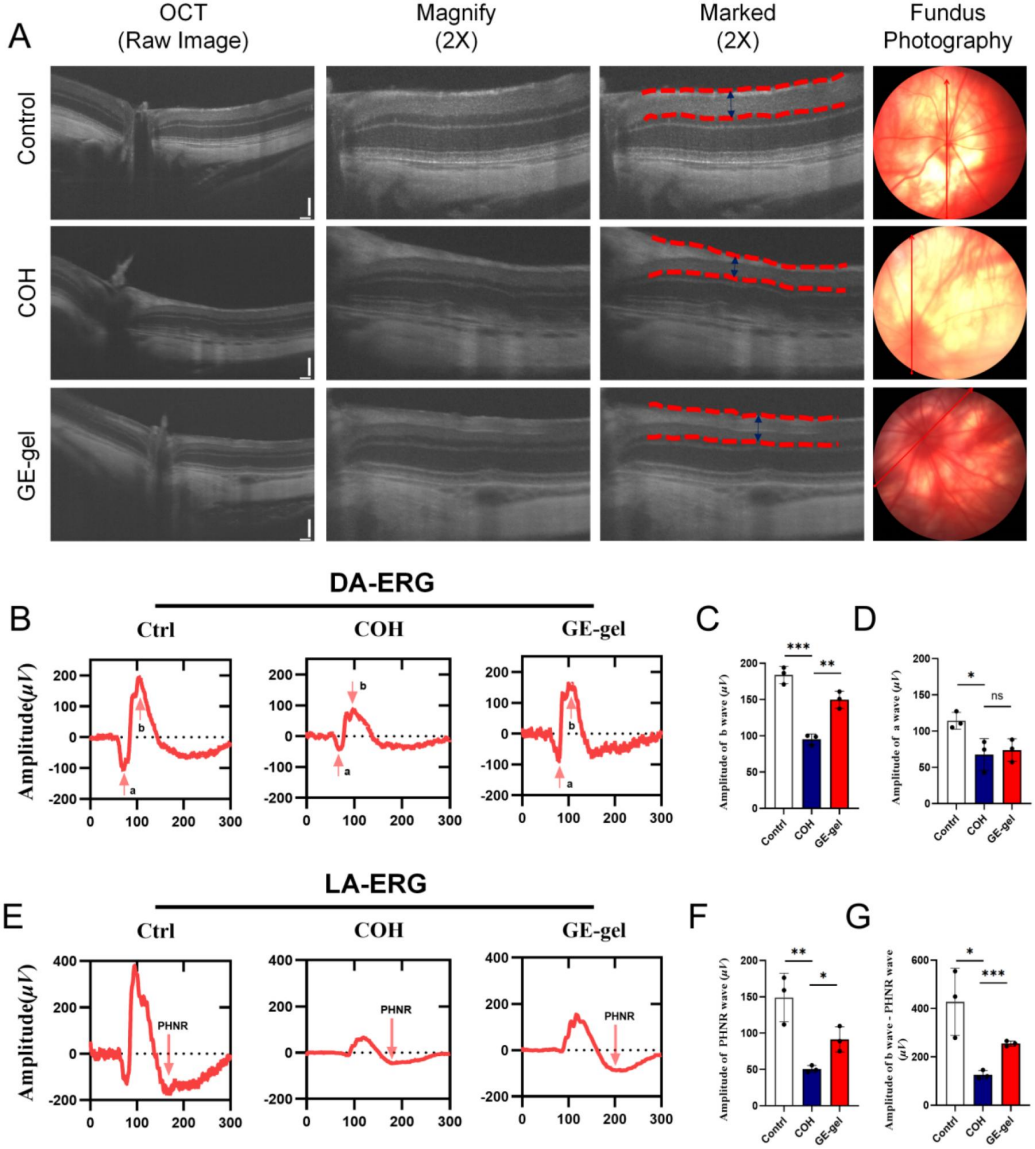
*In vivo* ophthalmic evaluation of GE-gel. (**A**) OCT and fundus images of rat eyes, red dashed lines indicate the nerve fiber layer (NFL), GCL and IPL. (**B**) Dark-adapted electroretinography (DA-ERG) for assessment of retinal electrophysiological function. (**C**) b-wave amplitude in DA-ERG. (**D**) a-wave amplitude in DA-ERG. (**E**) Light-adapted electroretinography (LA-ERG) for assessment of retinal electrophysiological function. (**F**) Amplitude of the PhNR in LA-ERG. (**G**) PhNR/b-wave ratio in LA-ERG.

We used clinically relevant *in vivo* imaging and functional testing to do a non-invasive, dynamic assessment of how well the treatment worked. OCT showed that the inner retinal layers (RNFL, GCL and IPL) were much thinner in the COH group. This is a marker of structural damage in glaucoma (Figure 6A). GE-gel treatment efficiently maintained inner retinal thickness, similar to the control group, visually substantiating its capacity to impede or avert structural development *in vivo*. Fundus photography revealed no treatment-related anomalies, indicating the superior intraocular biocompatibility of GE-gel.

ERG was performed to evaluate retinal functional changes following treatment. Dark-adapted ERG responses (Figure 6B), which mainly reflect the activity of photoreceptors and bipolar cells in the outer retina, were markedly reduced in COH rats, as evidenced by decreased amplitudes of both the a-waves and b-waves (Figure 6C and D). Administration of GE-gel significantly increased the b-wave amplitude, suggesting improved functional integrity of bipolar cells and Müller glia within the inner retinal circuitry. A-wave amplitude did not reach statistical significance after treatment. The photopic negative response (PhNR), a component closely associated with RGC activity and downstream signaling pathways, was markedly diminished in the COH group. In contrast, GE-gel treatment preserved PhNR amplitude to a significant extent (*P* < 0.001, Figure 6E–G), consistent with the RGC preservation observed in Brn3a immunostaining. These electrophysiological findings indicate that GE-gel not only maintains RGC structural integrity but also supports functional signal transmission within the retinal network. While our 4-week study demonstrated good intraocular biocompatibility based on fundus photography and retinal histology, long-term safety beyond 1 month has not been evaluated. Potential concerns include chronic foreign body reaction, accumulation of degradation products or late-onset inflammation. Future studies should include extended observation periods (e.g. 3–6 months) with comprehensive ocular and systemic safety assessments before clinical application.

Compared with other IOP-independent neuroprotective strategies, GE-gel offers several distinct advantages. Neurotrophic factor delivery (e.g. BDNF) faces challenges of rapid degradation and poor penetration, requiring repeated injections or viral vectors with safety concerns. Stem cell therapies hold regenerative potential but raise concerns regarding tumorigenicity, immune rejection and ethical hurdles. In contrast, GE-gel utilizes plant-derived exosomes with low immunogenicity, a biocompatible fibrin matrix that avoids foreign body reactions and a dual mechanism of antioxidant and immunomodulatory actions without introducing live cells. Thus, GE-gel represents a safe, off-the-shelf and sustained neuroprotective platform.

### RNA-Seq analysis of rat retina

**Figure 7:**
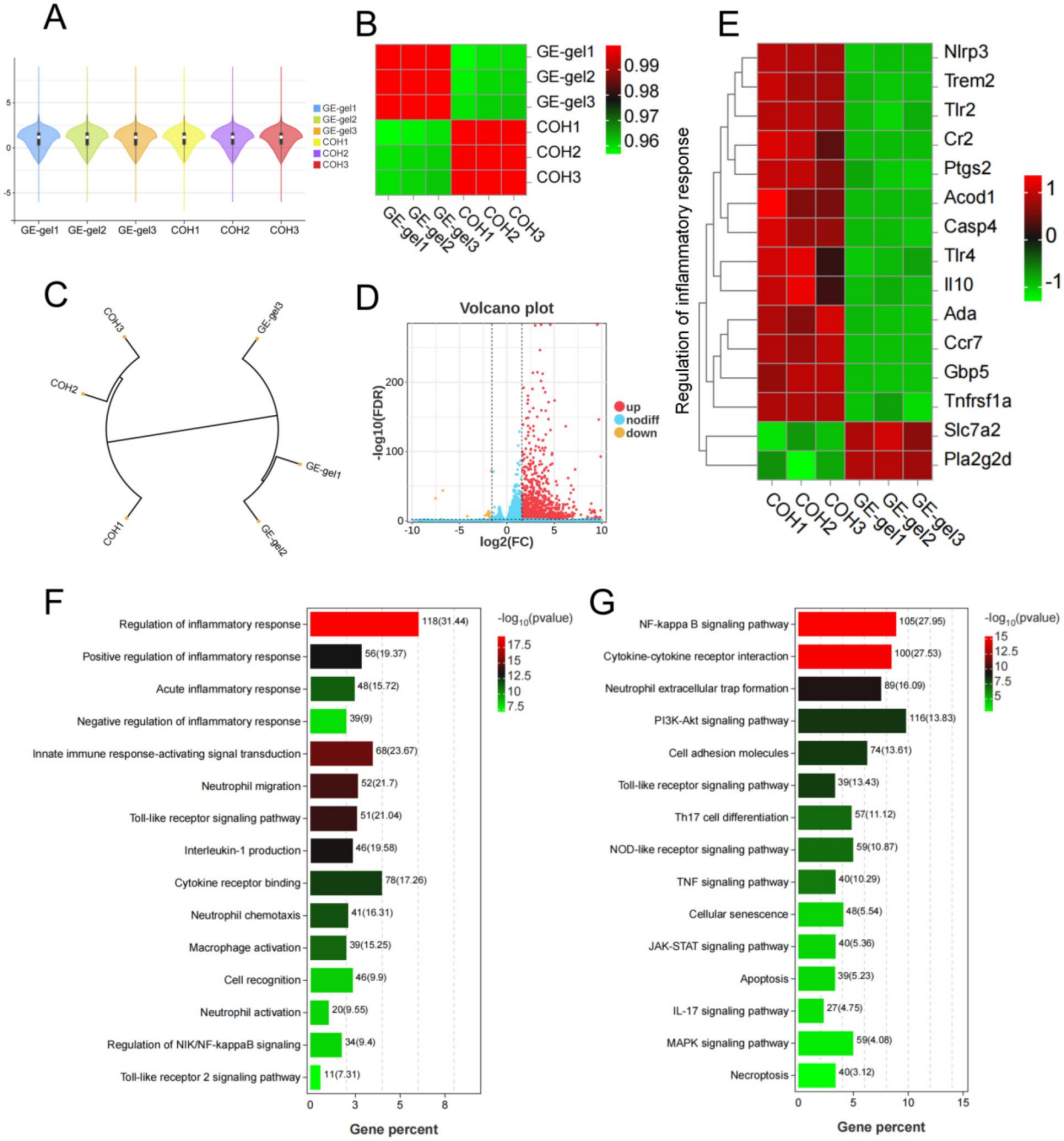
RNA-seq analysis of gene expression in rat retinal tissues. (**A**) Violin plots showing gene expression distribution in the COH and GE-gel groups. (**B**) Heatmap assessing intra-group reproducibility and inter-group differences in gene expression profiles. (**C**) Hierarchical clustering dendrogram of the COH and GE-gel groups. (**D**) Volcano plot depicting differentially expressed genes between the COH and GE-gel groups. (**E**) Heatmap of inflammation-related differentially expressed genes between the COH and GE-gel groups. (**F**) GO enrichment analysis of differentially expressed genes between the COH and GE-gel groups. (**G**) KEGG pathway enrichment analysis of differentially expressed genes between the COH and GE-gel groups.

To elucidate the molecular mechanisms underlying the neuroprotective effects of GE-gel *in vivo*, retinal tissues from COH model rats and GE-gel-treated rats were examined through RNA-seq. This study aimed to delineate transcriptional modifications on a genome-wide scale and to identify critical signaling pathways associated with therapeutic intervention. Quality checks on the sequencing data showed that the distributions of gene expression were very similar between biological replicates within each group (Figure 7A). Figure 7B and C show that correlation heatmaps and hierarchical clustering analysis were able to tell the COH and GE-gel groups apart. This proves that the data is reliable and shows that there are big differences in the transcriptomes of the two groups. A total of 12 300 genes were identified across both retinal groups, with 996 genes uniquely expressed in the COH group and 163 in the GE-gel group (Supplementary Figure S8). Figure 7D shows the results of a study on differential gene expression. It reveals that there were numerous changes in the transcription of genes in the GE-gel group compared to the COH group. Most of the genes were downregulated. This general trend suggests that the main reason GE-gel works as a treatment may be that it stops a lot of gene networks from being activated in the wrong way when someone is sick.

We focused on our primary hypothesis concerning the anti-inflammatory effect by examining genes associated with the ‘Regulation of inflammatory response’ gene set. The heatmap analysis showed that many important pro-inflammatory mediators, such as Nlrp3, Trem2, Tlr2, Tlr4 and Casp4, were less active in the GE-gel group (Figure 7E). The genes represent distinct inflammatory pathways. Nlrp3 encodes a key component of the NLRP3 inflammasome, Trem2 is involved in microglial activation and phagocytosis, Tlr2 and Tlr4 are pattern recognition receptors that sense damage-associated molecular patterns and initiate NF-κB signaling and Casp4 mediates non-canonical inflammasome activation and inflammatory cell death [36–39]. These results furnish molecular-level proof that GE-gel has anti-inflammatory properties by targeting and inhibiting various classical inflammatory molecules in the retina. Additionally, Western blot analysis was performed to confirm the expression of M1 microglia-associated proteins (NLRP3, TLR2 and Ptgs2) in the BV2 microglial cell culture. Figure S9 shows that GE-gel lowered the levels of NLRP3, TLR2 and Ptgs2, which means that it stopped M1 polarization. Subsequent Gene Ontology (GO) enrichment analysis indicated that differentially expressed genes were enriched in biological processes, including ‘regulation of inflammatory response’, ‘positive regulation of inflammatory response’ and ‘acute inflammatory response’ (Figure 7F). These findings support the downregulation of pro-inflammatory genes and suggest that GE-gel affects the recruitment of immune cells. The Kyoto Encyclopedia of Genes and Genomes (KEGG) pathway enrichment analysis identified critical signaling pathways influenced by GE-gel therapy, such as the ‘NF-kappa B signaling pathway’, ‘TNF signaling pathway’ and ‘apoptosis’ (Figure 7G).

### scRNA-seq analysis

**Figure 8:**
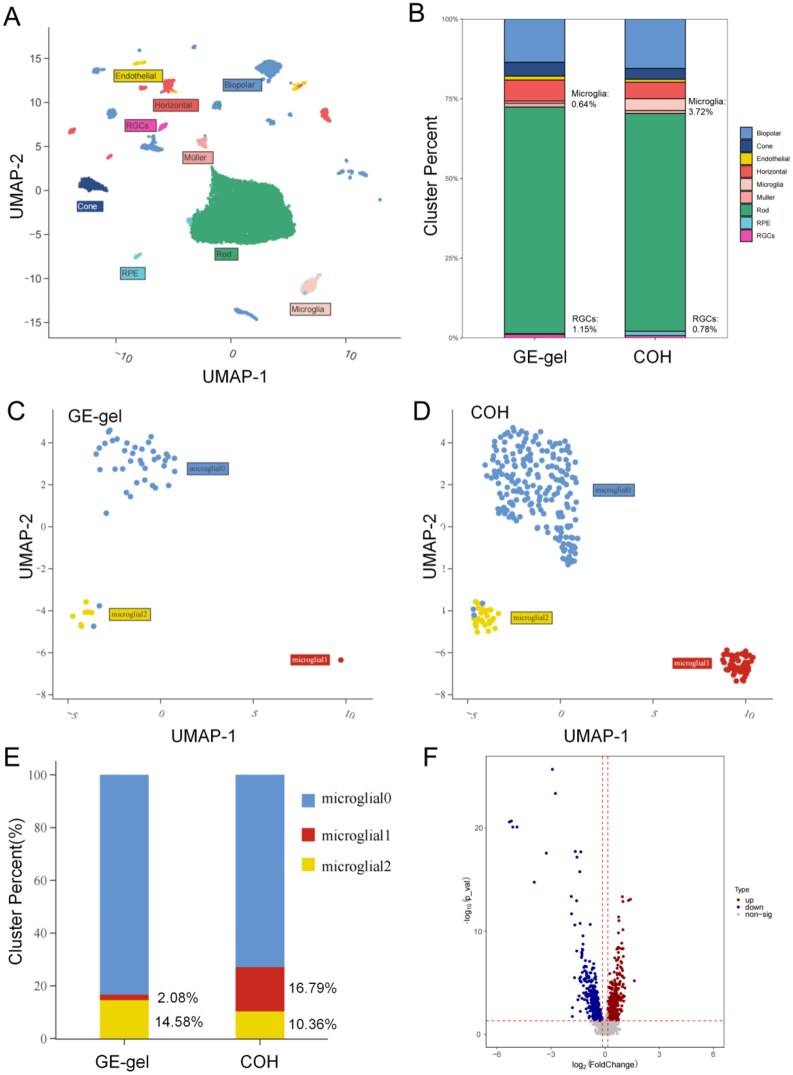
Single-cell transcriptomic analysis of retinal cell populations. (**A**) Identification of nine major retinal cell types. (**B**) Proportional distribution of the nine identified retinal cell types in the GE-gel and COH groups. (**C** and **D**) Subcluster classification of retinal microglia in the GE-gel and COH groups. (**E**) Proportional distribution of retinal microglia subpopulations. (**F**) Volcano plot showing differentially expressed genes in retinal microglia between the COH and GE-gel groups.

Even while bulk transcriptome sequencing showed changes in molecules all over the body, the very different types of cells in the retina meant that we needed single-cell resolution to figure out how the treatment worked on different types of cells. scRNA-seq was conducted to ascertain the cell types targeted by GE-gel. Using UMAP-based clustering, we found nine main categories of retinal cells, such as RGCs, different types of neurons, glial cells and endothelial cells (Figure 8A). The analysis of cell-type proportions showed that there were markedly more microglia in the COH group (3.72%) than in the GE-gel group (0.64%) (Figure 8B). This means that GE-gel helps reduce the number of immune cells in the retina and reduces chronic inflammation.

Further subclustering of microglia identified three functionally distinct subpopulations (Figure 8C and D), with Microglia1 representing pro-inflammatory M1-like microglia and Microglia2 representing anti-inflammatory M2-like microglia. As shown in Figure 8E, the pro-inflammatory Microglia1 subpopulation accounted for 16.79% in COH retinas, which sharply decreased to 2.08% following GE-gel treatment. Concurrently, the proportion of Microglia2 was maintained at a higher level. These findings provide single-cell resolution evidence that GE-gel selectively suppresses harmful pro-inflammatory microglia while preserving or promoting anti-inflammatory/stable subpopulations. This observation aligns with and extends prior *in vitro* flow cytometry and *in vivo* immunofluorescence data regarding M1/M2 polarization.

Differential gene expression profiling of retinal microglia between the COH and GE-gel groups further substantiated the extensive suppression of pro-inflammatory transcriptional patterns post-treatment (Figure 8F). In conjunction with subpopulation dynamics, GE-gel seems to modulate microglia-mediated neuroinflammation via a dual mechanism of diminishing overall microglial density in the retina and reprogramming remaining microglia from a pro-inflammatory Microglia1-dominated phenotype to a balanced or reparative state. This specific immunomodulatory mechanism, elucidated at single-cell resolution, highlights microglia as a principal cellular target of GE-gel and enhances the comprehension of its anti-inflammatory effects from protein-level indicators to transcriptome regulation.

## Conclusion

In this study, we developed and systematically validated an injectable GE-gel delivery system for precise modulation of glaucoma-associated neuroinflammation and protection of RGCs. Encapsulation of GE within the 3D porous network of fibrin gel effectively overcame the challenges of rapid clearance and short intravitreal retention of exosomes, achieving sustained local release. Mechanistically, GE-gel exerts neuroprotective effects through both cellular and immunomodulatory behaviors. The GE-gel could mitigate oxidative stress-induced ROS accumulation and inhibit RGC apoptosis and could suppress M1 microglial polarization while promoting M2 anti-inflammatory polarization, thereby remodeling the retinal immune microenvironment and interrupting the inflammation-oxidative stress-neuronal death pathological feedback loop. In the COH rat model, GE-gel treatment reduced retinal neuroinflammation, maintained RGC counts and laminar architecture and reinstated RGC-associated electrical function. scRNA-Seq demonstrated a reduction in the proportion of pro-inflammatory Microglia1 cells and an increase in the proportion of anti-inflammatory Microglia2 cells, thereby validating the reprogramming of microglial functional states. By targeting microglial polarization, GE-gel provides an applicable way to protect RGCs from damage in glaucoma without relying on IOP.

## Supplementary Material

rbag146_Supplementary_Data
