# Supplementary material for: Ginseng-derived exosomes loaded in fibrin gel promote retinal ganglion cell survival in glaucoma by exerting anti-inflammatory effects through modulating microglial polarization
Source: Regen Biomater. 2026 Jul 10;13:rbag146. doi: 10.1093/rb/rbag146 (PMC13420499; doi:10.1093/rb/rbag146)
Supplement: rbag146_Supplementary_Data [file rbag146_supplementary_data.docx]

**Supplementary Information**

**Ginseng-derived exosomes loaded in fibrin gel promote retinal ganglion cell survival in glaucoma by exerting anti-inflammatory effects through modulating microglial polarization**

Dengming Zhou^1, 2#^, Dehong Tan^3, 4#^, Xuanqi Peng^5^, Cailian Fang^6^, Yongzhen Yu^1,2^, Haroon Iqbal^1, 2^, Junfan Zhang^1, 2^, Lin Fu^1, 2^, Lewei Tang^1,2^, Xiaoyu Zhou^3, 4^, Xinyue Zhang^3, 4^, Run Xiao^6^, Wenxiang Zhu^3, 4*^, Ludan Yue^5*^, and Yuanbo Liang^1, 2*^

^1^ State Key Laboratory of Ophthalmology, Optometry and Visual Science, Eye Hospital, Wenzhou Medical University, Wenzhou, 325027, China.

^2^ Eye Research Center, Hangzhou Institute of Medicine, Chinese Academy of Sciences, Eye Hospital, Wenzhou Medical University, Hangzhou, 310018, China.

^3^ Aier Academy of Ophthalmology, Central South University, Changsha, 410083, China.

^4^ Aier Glaucoma Institute, Changsha Glaucoma Diagnosis and Treatment Technology Innovation Center, Changsha Aier Eye Hospital, Changsha, 410015, China.

^5^ Department of Biochemistry and Molecular Biology, School of Medicine, Southeast University, Nanjing 210009, China

^6^ School of Molecular Medicine, Hangzhou Institute for Advanced Study, UCAS, Hangzhou, Zhejiang 310024, China

^#^Contributed equally to this work.

**Corresponding Authors:** wenxiang@hnu.edu.cn (W. Zhu), yueld@seu.edu.cn (L. Yue), yuanboliang@126.com (Y. Liang)

**Supplementary Method**

**Protein Extraction and Western Blotting**

Total protein from BV2 microglia was isolated using RIPA lysis buffer (Beyotime, P0013C) containing 1 percent PMSF (Servicebio, G2008-1ML) and 1X protease/phosphatase inhibitors (Beyotime, P1045). Cells were washed twice with chilled PBS, lysed in the complete medium on ice for 30 min, and centrifuged at 12,000 rpm (4 ^o^C) for 15 min to remove debris. The protein-rich supernatant was mixed with 5X sample loading buffer (ABclonal, RM00001), denatured at 95 ^o^C for 10 min, and stored at -80 ^o^C.

Electrophoresis was performed using precast gels (ACE Biotechnology, X12012LGel) in 1 L rapid running buffer at 180 V for ~60 min, loading 5 microliters of markers and samples per lane. Proteins were transferred onto methanol-activated PVDF membranes (7 cm × 9 cm) within a rapid transfer tank (constant current of 300 mA for 30 min). The transfer sandwich was built sequentially from cathode to anode: negative plate, sponge, filter paper, gel, membrane, filter paper, sponge, and positive plate.

Following a 10-min blocking step and three TBST rinses, membranes underwent overnight incubation at 4 ^o^C with primary antibodies against TLR2 (1:1000, ABclonal, A19125), NLRP3 (1:2000, Proteintech, 30109-1-AP), PTGS2 (1:2000, Proteintech, 27308-1-AP), and beta-actin (1:10000, ABclonal, AC038). After three 10-min TBST washes, membranes were incubated with HRP-Goat Anti-Rabbit Recombinant Secondary Antibody (1:5000, Proteintech, RGAR001) for 2 h at room temperature. Blots were washed four times with TBST (10 min each), visualized with HRP substrates (1-2 min) on a gel imaging system, and quantified using ImageJ software by normalizing band densities to beta-actin.

**In Vivo Tracking of GE**

For in vivo tracking, GE were labeled with DiO (green fluorescent lipophilic dye) following the same protocol as DiI labeling. Briefly, GE (30 μg/mL) were incubated with 10 μM DiO at 37°C for 30 min, washed twice with PBS by ultracentrifugation (100,000 × g, 60 min), and resuspended in PBS. For the GE-gel group, DiO-labeled GE were incorporated into the fibrin gel as described above. Rats received a single intravitreal injection of DiO-labeled GE (5 μL, 30 μg/mL) or DiO-labeled GE-gel. At days 14 post-injection, animals were euthanized, eyes were enucleated and fixed in 4% PFA, and retinas were dissected, flat-mounted on slides with anti-fade mounting medium containing DAPI. Fluorescence images were captured using a confocal microscope with identical acquisition settings across groups.

**Supplementary Data**


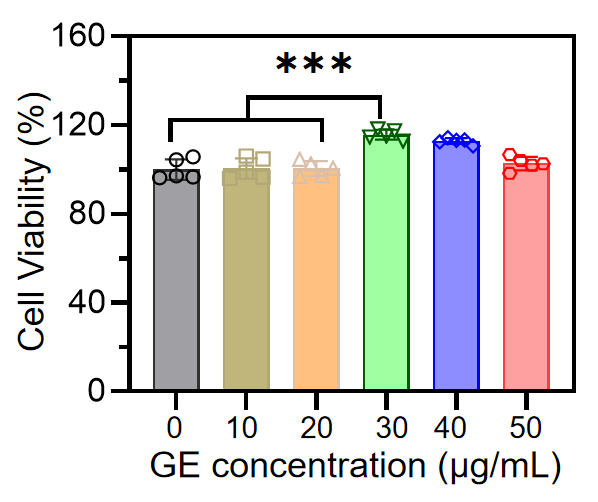


**Figure S1.** CCK-8 cell viability assay at different concentrations of GE.


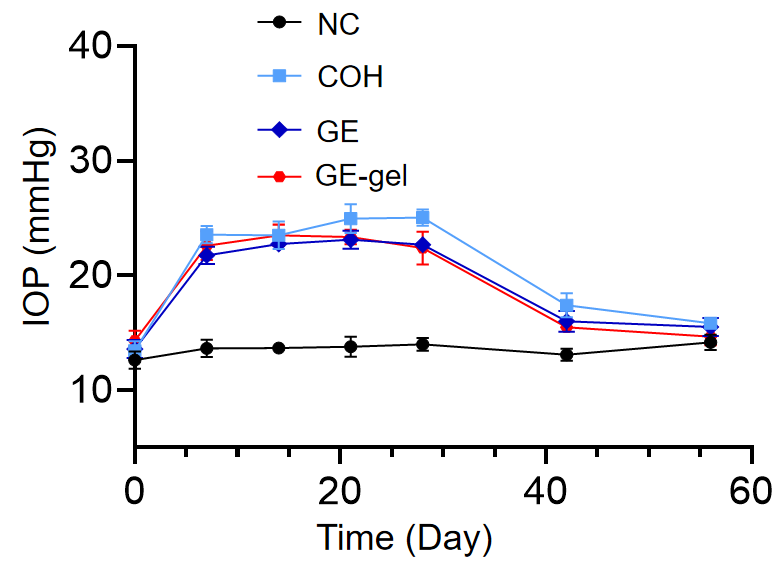


**Figure S2.** Statistical analysis of intraocular pressure in the right eyes of rats across different treatment groups.


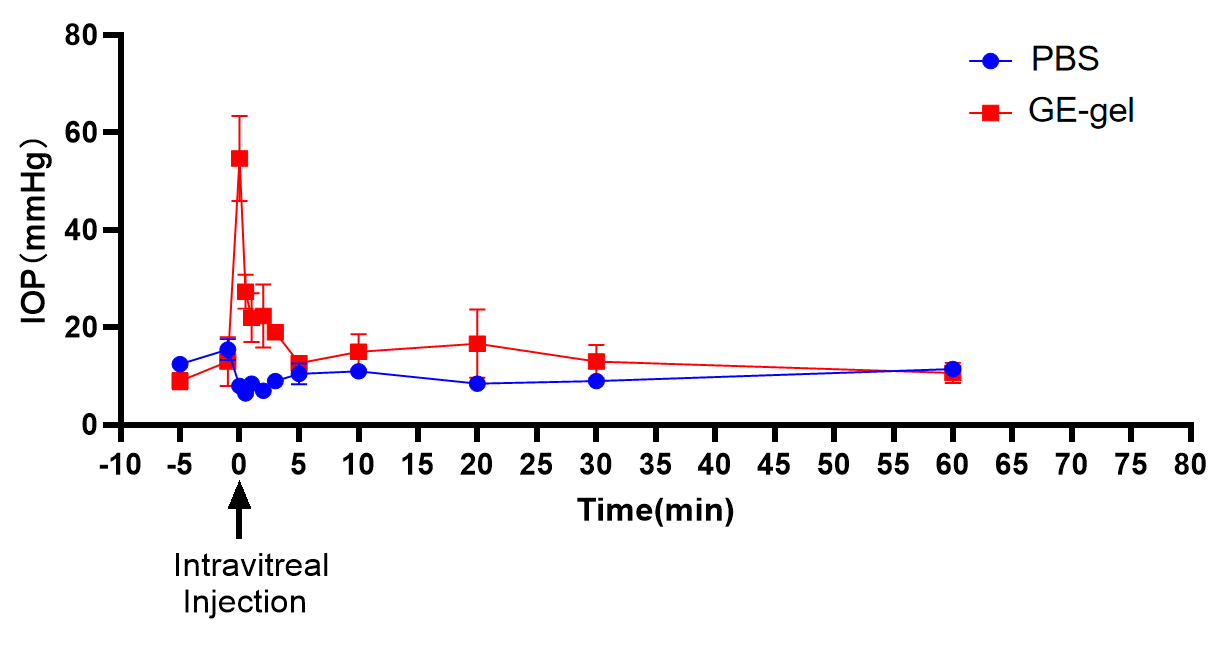


**Figure 3.** Intraocular pressure changes in rats after intravitreal injection of 5 μL PBS or GE-gel.


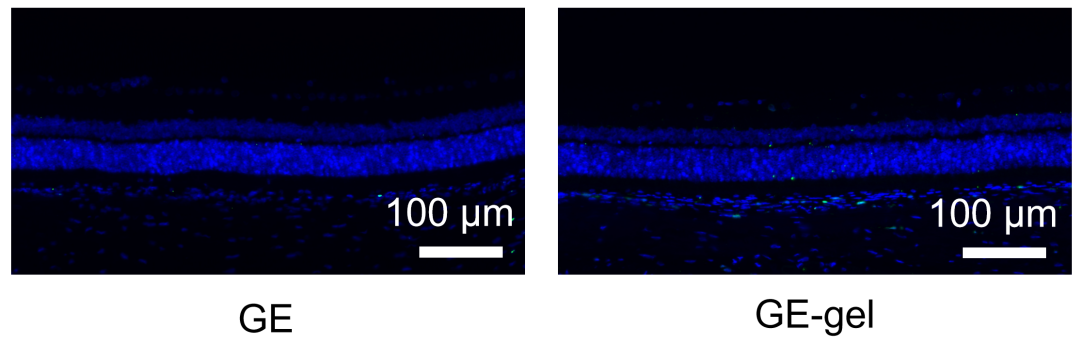


**Figure S4.** *In vivo* retinal release of GE and GE-gel following intravitreal injection (DiO-labeled GE).


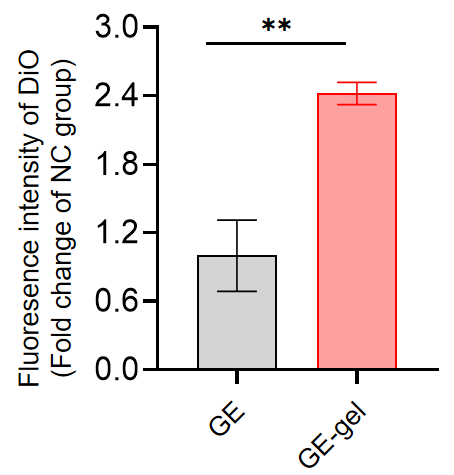


**Figure S5.** Quantitative fluorescence analysis of DiO-labeled GE release in the retina in vivo.


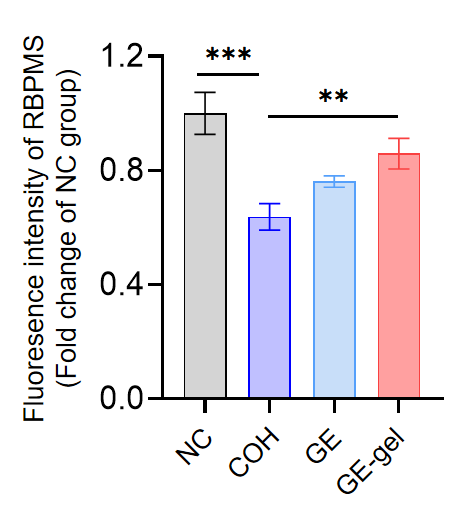


**Figure S6.** Quantitative analysis of RBPMS immunofluorescence staining in the retina in vivo.
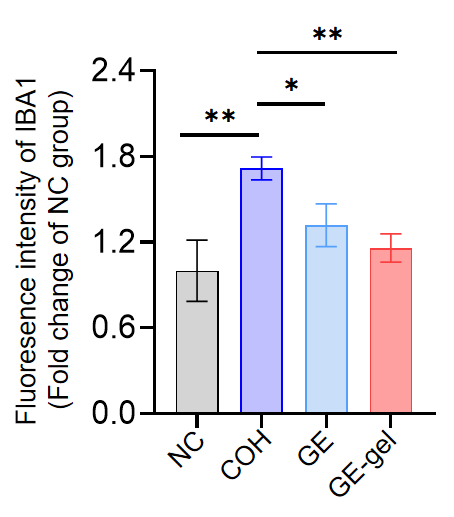


**Figure S7.** Quantitative analysis of IBA1 immunofluorescence staining in the retina in vivo.
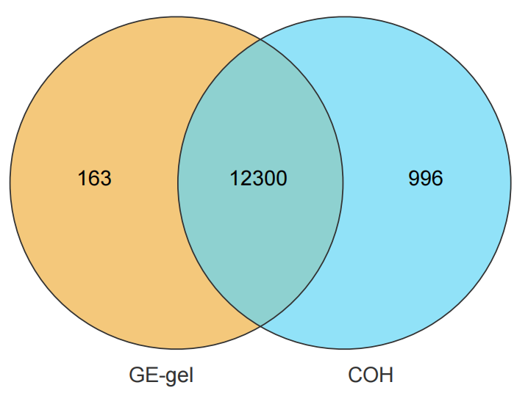


**Figure S8.** Venn diagram showing the overlap of expressed genes between the COH (control) group and the GE-gel group.


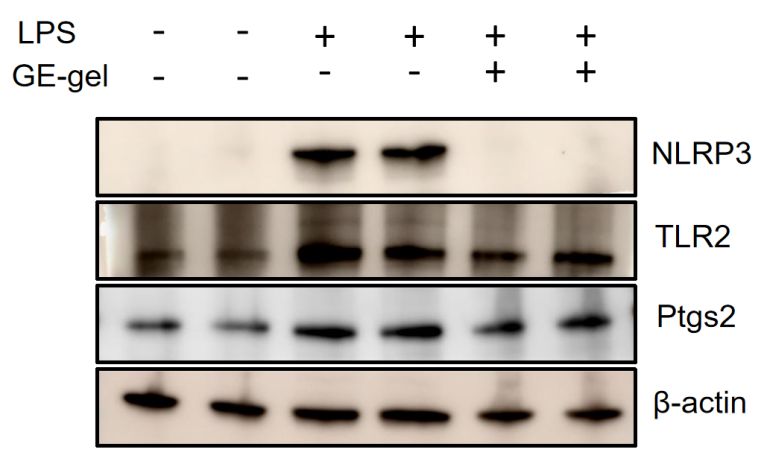


**Figure S9.** Western blot analysis validating the expression levels of proteins (NLRP3, TLR2, and Ptgs2) in the BV2 microglial cell model.
